# Subtypes of avoidant/restrictive food intake disorder in children and adolescents: a latent class analysis

**DOI:** 10.1016/j.eclinm.2024.102440

**Published:** 2024-02-01

**Authors:** Javier Sanchez-Cerezo, Josephine Neale, Nikita Julius, Tim Croudace, Richard M. Lynn, Lee D. Hudson, Dasha Nicholls

**Affiliations:** a Division of Psychiatry, Department of Brain Sciences, Imperial College London, London, UK; b Department of Psychiatry, Hospital Universitario Puerta de Hierro, Majadahonda, Madrid, Spain; c Priory Hospital Ticehurst House, Ticehurst, East Sussex, UK; d School of Health Sciences, University of Dundee, Dundee, UK; e Institute of Child Health, University College London, London, UK

**Keywords:** ARFID, Eating and feeding disorders, Children and adolescents, Latent class analysis, ASD

## Abstract

**Background:**

The Diagnostic and Statistical Manual of Mental Disorders, fifth edition (DSM-5) describes three primary avoidant/restrictive food intake disorder (ARFID) subtypes including sensory sensitivity, lack of interest in food or eating, and fear of aversive consequences. Studies exploring these subtypes have yielded varying results. We used latent class analysis (LCA) based on the psychopathology of ARFID in a sample of children and adolescents to empirically identify classes.

**Methods:**

We carried out a surveillance study of ARFID in collaboration with the British Paediatric Surveillance Unit (BPSU) and the Child and Adolescent Psychiatry Surveillance System (CAPSS) in the United Kingdom and the Republic of Ireland from 1st of March 2021 to 31st of March 2022. Paediatricians and child and adolescent psychiatrists were contacted monthly to report newly diagnosed cases of ARFID electronically and complete a detailed clinical questionnaire. Cases aged 5–18 years were included. LCA was performed specifying 1–6 classes and likelihood-based tests for model selection. The Bayesian Information Criterion (BIC), the Akaike Information Criterion (AIC) and the Sample-Size Adjusted BIC were used to determine the most parsimonious model. Analysis of variance (ANOVA) and χ2 tests were used to compare the characteristics of the identified classes. A multinomial logistic regression (MLR) was performed to investigate predicting factors for the latent classes.

**Findings:**

We identified 319 children and adolescents with ARFID. LCA revealed four distinct classes which were labelled as *Fear subtype*, *Lack of Interest subtype*, *Sensory subtype*, and *Combined subtype*. The probability of being classified as these were 7.2% (*n* = 23), 25.1% (*n* = 80), 29.5% (*n* = 94) and 38.2% (*n* = 122), respectively. Age at diagnosis, sex, weight loss, distress associated with eating, and autism spectrum disorder diagnosis were identified as predictors of class membership.

**Interpretation:**

LCA identified four different classes in a sample of children and adolescents with ARFID. The *Combined Subtype*, a mixed presentation was the most common. The other three classes resembled the subtypes described in the literature. Clinicians should be aware of these different presentations of ARFID as they may benefit from different clinical interventions.

**Funding:**

This study was funded by the Former EMS Ltd (charity number 1098725, registered October 9th 2017).

**Research in context**

**Evidence before this study**

The Diagnostic and Statistical Manual of Mental Disorders, fifth edition (DSM-5) describes three primary avoidant/restrictive food intake disorder (ARFID) subtypes including sensory sensitivity, lack of interest in food or eating, and fear of aversive consequences which need testing in clinical populations of children and adolescents. We searched for articles published from January 1st, 2013 to May 31st, 2023 using the terms “ARFID” and “avoidant restrictive food intake disorder” in Embase, Medline and PsycINFO with no language restrictions. Prior research exploring the ARFID subtypes has relied mostly on retrospective chart review methodology and empirical data exploring the distinct subtypes remains limited.

**Added value of this study**

We used latent class analysis (LCA) solely based on the psychopathology of the proposed DSM-5 ARFID subtypes in a large representative sample of children and adolescents ascertained through active surveillance. Four classes of children and adolescents with ARFID were identified which had different clinical and demographic characteristics. Our study showed that the most common presentation of ARFID in children and adolescents presenting to paediatricians and child and adolescent psychiatrists was a combination of the sensory sensitivity and the lack of interest in food or eating subtypes whereas the least common was the fear of aversive consequences subtype.

**Implications of all the available evidence**

Our surveillance study provides evidence that children and adolescents with ARFID attending secondary care most often present with sensory sensitivities, lack of appetite, or a combination of these two. Due to the heterogeneity of clinical presentations, children and adolescents with ARFID should be assessed and treated by a multidisciplinary team that ensures patients’ needs are recognised. The different characteristics of each subtype of ARFID suggest clinicians should identify the class early on in treatment in order to provide the most appropriate treatment and identify common comorbidities.

## Introduction

Avoidant/restrictive food intake disorder (ARFID) is characterised by persistent disturbance in feeding or eating which results in inability to meet nutritional and/or energy needs leading to at least one of the following: weight loss or failure to achieve appropriate weight gain; nutritional deficiency; dependence on enteral feeding or nutritional supplements; or significant interference with psychosocial functioning. ARFID is not associated with concerns about gaining weight nor with a preoccupation about body weight, shape, or size.^1^^,^^2^ A recent systematic review of epidemiological studies on ARFID in children and adolescents found highly heterogeneous estimates of prevalence, ranging from 0.3 to 64% depending on the study setting, methodology employed or sample characteristics.^3^ The only ARFID incidence study to date, a surveillance study conducted in Canada, reported an incidence of 2.02 per 100,000 young people aged 5–18 years presenting to paediatricians.^4^

The Diagnostic and Statistical Manual of Mental Disorders (5th ed.; DSM-5)^1^ and the International Classification of Diseases (11th ed.; ICD-11)^2^ describes three subtypes of food restriction in ARFID: sensitivity to sensory aspects of food; a lack of interest in food or eating; or fear of aversive consequences associated with eating. However, there remains a lack of empirical data supporting validity of these subtypes. In a Swiss school-based population study, 3.2% of children reported features of ARFID. Of these, 39% indicated lack of interest in eating or food, 60% had sensory sensitivities, and 15% avoided food due to negative consequences. An additional 15% exhibited a mixed presentation of at least two of the ARFID subtypes.^5^ Other studies have reported that a mixed presentation was present in more than 50% of their samples.^6^, ^7^, ^8^

Sex and age differences in these presentations are still not clearly understood. A surveillance study in Canada revealed that males with ARFID exhibit greater sensory sensitivity compared to females^4^ and Zickgraf et al. (2019) suggested that it is more common in younger than older children.^9^ By contrast, patients with concern about aversive consequences are reported to be more often female than male.^10^ However, a recent cross-sectional study with 261 patients with ARFID aged 2–17 years found that the ARFID subtypes were not associated with patient sex.^11^

Young people with ARFID often present with a comorbid psychiatric disorder or medical conditions. Anxiety disorders are the most common psychiatric comorbidity, with estimates ranging from 9.1% to 72%.^3^ Kambanis et al. (2020)^12^ found that the fear of aversive consequences and the sensory sensitivities subtypes were associated with a higher likelihood of comorbid anxiety, obsessive-compulsive, and trauma-related disorders. Other common comorbidities with ARFID are neurodevelopmental disorders, especially autism spectrum disorder (ASD).^3^ Children and adolescents with ASD can present with any of the three ARFID subtypes^13^ but it has been described that they showed more sensory sensitivities and greater lack of interest in eating than individuals without ASD.^11^

From a weight status standpoint, ARFID is a very heterogeneous disorder.^14^ While individuals who restrict their food intake due to fear of aversive consequences or lack of interest are underweight on average,^15^ others who limit their food variety because of sensory sensitivities have weights across the weight spectrum.^9^

Latent class analysis (LCA) is a statistical procedure that can be used to identify and describe heterogeneity within a population via a model-based cluster analysis approach.^16^ This method is frequently applied when responses are available from focussed set of categorical indicator variables.^17^ Katzman et al.^18^ used LCA in a sample of children and adolescents with ARFID ascertained through active paediatric surveillance in Canada using a combination of indicator variables based on i) ARFID diagnostic criteria, ii) sign and symptoms, iii) hospitalizations, and iv) autism spectrum disorder (ASD) comorbidity. Results identified three subgroups: lack of appetite sensory sensitivity, and ‘acute medical’, characterised by medical hospitalisation. Although a 3-class model was best fit, 14.5% of cases presented with a mixture of acute medical and lack of appetite, being assigned to a fourth group.

The aim of the current study was to identify ARFID classes using LCA based on the psychopathology rather than service utilisation and other features and to investigate predictors of class membership using multinomial logistic regression (MLR). Data were from a large national sample of children and adolescents with ARFID accessing secondary care, reported by both paediatricians and child and adolescent psychiatrists in the United Kingdom (UK) and the Republic of Ireland (ROI). We hypothesised that the LCA clustering approach would successfully identify different subtypes (classes) of ARFID in children and adolescents but were agnostic about what number of classes would be the best fit. We also hypothesised that sex, age, and comorbidities would predict class membership.

## Methods

### Design

An observational, surveillance study was undertaken in collaboration with the British Paediatric Surveillance Unit (BPSU) and the Child and Adolescent Psychiatry Surveillance System (CAPSS).^19^ These surveillance systems work by sending monthly electronic reporting cards listing health conditions under study to all consultant paediatricians (BPSU) and consultant child and adolescent psychiatrists (CAPSS) in the UK and ROI. Reporting cards are returned to the surveillance office, who inform the research team when a clinician reports a case. Clinicians are also asked to report if they have not seen any cases. A committee-approved, study-specific questionnaire is then sent to the reporting clinician for further details about the case. Reporting clinicians were asked to report patients’ sex in the questionnaire.

### Ethics

The study protocol was approved by both BPSU and CAPSS Executive Committees. Ethical approval was obtained from West Midlands—Black Country Research Ethics Committee (Integrated Research Application System ID 273665; REC 20/WM/0256). Due to the nature of the study, patient and parental consent was not required. Data were collected in England and Wales following Section 251 advice from the Confidentiality Advisory Group of the Health Research Authority (20/CAG/0120). Data were collected in Scotland following advice from the Public Benefit and Privacy Panel for Health and Social Care (HSC-PBPP) (2021-0113). Northern Ireland Privacy Advisory Committee requirements were met to collect data.

### Data collection

Newly diagnosed cases of ARFID attending secondary care were ascertained across the UK and ROI over a 13-month period (1st of March 2021 to 31st of March 2022). Cases were reported based on a broad definition from DSM-5 diagnostic criteria (that can be seen in Table 1), then confirmed by the research team using more precise analytic case definition. Inclusion and exclusion criteria used by the research team are listed in Table 2. The research team discussed and agreed any cases where there was unclear inclusion or classification. When a questionnaire lacked sufficient information to confirm a case, the case was subsequently excluded.

**Table 1:** Instructions to clinicians for notification of potential cases.

Any child or adolescent aged 5–16 (5–18 for child and adolescent psychiatrists) years with persistent restriction of quantity and/or range of food intake, associated with one or both of the following: • Nutritional deficiency that requires additional clinical investigation or treatment (e.g., anaemia, micronutrient deficiency, weight loss or poor growth, reliance on nutritional supplementation) that is not fully accounted for by poverty or neglect, cultural practice or an existing medical condition or another mental disorder.^a^ • Interference with day-to-day functioning due to eating behaviour (e.g., unable to eat at school or with peers, needs to take preferred foods when out of home, extreme and frequent distress about eating). Not explained by ANY of the following: • Lack of available food (e.g., from poverty, famine, or neglect) • Culturally sanctioned practice (e.g., endorsed religious and cultural practice) • Other known diagnosis ○ e.g., Allergy to specific food group (e.g., dairy) ○ Gastrointestinal disorder ○ Constipation ○ Swallowing difficulties ○ Other eating disorder, e.g., anorexia nervosa, bulimia nervosa ○ Other medical or psychiatric disorder that fully explains food restriction (not requiring additional clinical attention), e.g., depression, anxiety, OCD, malignancy, diabetes mellitus, inflammatory bowel disease, thyroid disease.

a If the eating disturbance occurs in the context of another condition/disorder, then to meet case definition for ARFID, the severity of eating disturbance should exceed that routinely associated with the particular condition/disorder–and warrant additional clinical attention.

**Table 2:** Inclusion and exclusion criteria.

Any child or adolescent aged 5–18 years with persistent restriction of quantity and/or range of food intake, associated with one (or more) of the following:
Lack of appetite Lack of interest in food Logistics of feeding/eating behaviour not consistent with age and development (e.g., small bites/slow eating) Limited variety of food intake Rigid eating behaviour (e.g., brand-specific, food items on a plate cannot touch) Unfounded fear of aversive consequences of eating (e.g., fear of choking/vomiting)

And resulting in **ANY** one or more of the following bullet points:	
Anthropometric evidence of significant weight loss or growth impairment	As evidenced by any one of: • Weight-for-age <−2 SD from the international reference median value • Weight-for-height <−2 SD from the international reference median value • Height-for-age <−2 SD from the international reference median value • >10% body mass loss
Nutritional deficiency	As evidenced by any one of: • Absence (or near absence if other criteria definitely present) of entire food groups from diet (fruit and vegetables/carbohydrates and grains/protein/dairy products) • Nutritional blood investigation abnormalities (e.g., anaemia, micronutrient deficiency) • ≥50% daily caloric intake via prescribed nutritional or food supplementation • Use of any tube feeding not required by a concurrent medical condition.
Interference with psychosocial functioning	As evidenced by any one of: • Extreme and frequent distress about eating (tearfulness, tantrums, refusal to eat) • Inability to eat except only in certain situations (e.g., only alone/only with family members) • Other impairment of social and emotional development or functioning secondary to eating behaviour (e.g., poor school attendance, limited peer relationships, excessively long mealtimes impacting on self/family)
Not explained by **ANY** of the following:	
Lack of available food	e.g., from poverty, famine, or neglect
Culturally sanctioned practice	e.g., endorsed religious and cultural practice
Other known diagnosis	• Allergy to specific food group (e.g., dairy) • Gastrointestinal disorder • Constipation • Swallowing difficulties • Other eating disorder, e.g., anorexia nervosa, bulimia nervosa • Other medical or psychiatric disorder that fully explains food restriction (not requiring additional clinical attention) e.g., depression, anxiety, OCD, malignancy, diabetes mellitus, inflammatory bowel disease, thyroid disease.

OCD = obsessive compulsive disorder; SD = standard deviation.

Data on cases were entered by reporting paediatricians and psychiatrists and managed using Research Electronic Data Capture (REDCap),^20^ a secure, web-based software platform hosted at Imperial College London.

### Statistics

LCA was adopted to identify mutually exclusive ARFID latent classes. Six simple binary indicator variables for the analysis were chosen based on the criteria for diagnosis of ARFID in the DSM-5^1^ and on previous research.^15^ The indicator variables included in the LCA were lack of appetite, lack of interest in eating or food, difficulties with practicalities of feeding behaviours (e.g., small bites or slow eating), sensory sensitivity (e.g., taste, smell, colour, or texture), rigid eating behaviours (e.g., brand-specific or food items cannot touch in the plate), and fear of aversive consequences of eating (e.g., fear of choking or vomiting). Indicator variables were all coded as binary (0/1) variables. The indicators were individually reported on the questionnaires.

LCA was performed specifying 1–6 classes and likelihood-based tests for model selection. These included the Lo-Mendell-Rubin adjusted likelihood ratio test (LMR-LRT) and the bootstrapped likelihood ratio test (BLRT) which facilitate the assessment of whether adding a further class leads to a statistically significant improvement in LCA model fit. A non-significant *p* value for a *k* class solution thus lends support for the *k*−1 class solution.^21^ The most parsimonious number of latent classes was determined with reference to commonly reported information criteria that consider model parsimony in different ways, namely: Bayesian Information Criterion (BIC), Akaike Information Criterion (AIC) and Sample-Size Adjusted BIC. For all these models, lower values are favoured. Guidance indicates that BIC should be prioritized over AIC and Sample-Size Adjusted BIC in cases where model fit statistics are equivocal.^21^ In addition to global measures of model fit, bivariate standardised residuals were examined with values <|1.96| indicating conditional independence. Entropy values were also evaluated. Higher entropy denotes better class separation, with a value close to 1 being ideal.^22^

After determining the number of classes, individuals were assigned to a class based on their highest (posterior) probability. Average posterior probabilities (APPr) were calculated to evaluate the classification uncertainty for each class. An APPr is the average probability of an individual being assigned to a class given their scores on the indicator variables used to create the classes. Higher values (i.e., closer to 1.00) are desirable.^23^ The sample size in each class was considered in relation to guidance to avoid classes with less than 5% of the total sample.^23^ Analysis of variance (ANOVA) and χ^2^ tests were used to compare the characteristics of the identified classes. Post-hoc tests with Bonferroni correction were applied and adjusted residuals calculated to examine pair-wise differences if tests were significant. Finally, a MLR was performed to investigate predicting factors for the latent classes. Variables with statistically significant differences between classes in the univariate analyses were explored as covariates for the MLR. Due to sample size, variables with more than 10% of missing data were not used for the MLR. AIC and BIC were used to compare models including different covariates.^24^

Body mass index (BMI) was calculated and z-scores for height, weight and BMI were computed using UK 1990 growth reference data.^25^^,^^26^ Cut-off interpretations for BMI z-scores are as follows: Normal weight ≤+1 standard deviation (SD) to ≥−2 SD; thinness <−2 SD; severe thinness <−3 SD.^27^

Effect sizes for between-class differences were estimated with Cramer's φ_c_ or partial ɳ^2^, which can be interpreted as small (0.10 or 0.01), medium (0.30 or 0.06), or large (0.50 or 0.14), respectively.^28^ LCA was carried out using specialised procedures (syntax available) in software Mplus version 8.9.^29^ ANOVA and χ^2^ tests and MLR were conducted using Stata 17.^30^ Statistical significance was taken as a 2-sided *p* < 0.05.

### Role of funding source

The funding source had no role in study design, data collection, data analysis, data interpretation, or writing of the report.

## Results

### Case ascertainment and demographic characteristics

**Fig. 1:**
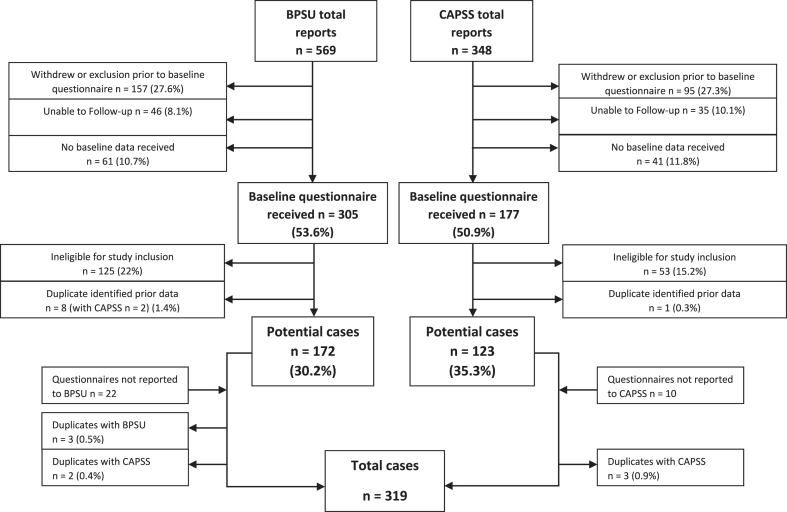
Flow diagram of case ascertainment. Figure 1 shows the flow of individuals from notification to case validation: after reporting a case to BPSU or CAPSS, clinicians were contacted to complete a questionnaire. Reporting errors (such as prevalent cases or confirmed diagnosis of anorexia nervosa) were excluded prior to baseline questionnaire completion after contacting the clinician. Unable to follow-up cases were those excluded due to clinicians stating that they did not wish to be included in the study (due to retirement, shortage of reporting capacity and so on). Cases were excluded if no response was obtained after multiple attempts to contact the notifying clinician or their team (no baseline data received). Completed questionnaires by reporting clinicians were examined to confirm cases were eligible for inclusion. Duplicates were identified and excluded. Additional cases from other sources that met inclusion criteria were added. BPSU = British Paediatric Surveillance Unit; CAPSS = Child and Adolescent Psychiatry Surveillance System.

BPSU surveyed 4298 consultant paediatricians and CAPSS surveyed 695 consultant child and adolescent psychiatrist during the study period. The response rate to the monthly electronic reporting cards was 78.5% for BPSU and 47.5% for CAPSS. A total of 917 potential cases of ARFID were reported (569 from BPSU and 348 from CAPSS). Information to include or exclude cases was received for 81.2% of BPSU cases and 78.1% of CAPSS cases. Case ascertainment can be seen in Fig. 1.

A total of 319 children and adolescents with ARFID aged 5–18 years (mean age = 11.2 years [SD = 3.8; range 5.00–17.99]; female = 45.5%) living in the UK and ROI were included. The majority were of white ethnicity (n = 248, 77.7%).

### Latent class analysis: identification of ARFID subgroups

**Fig. 2:**
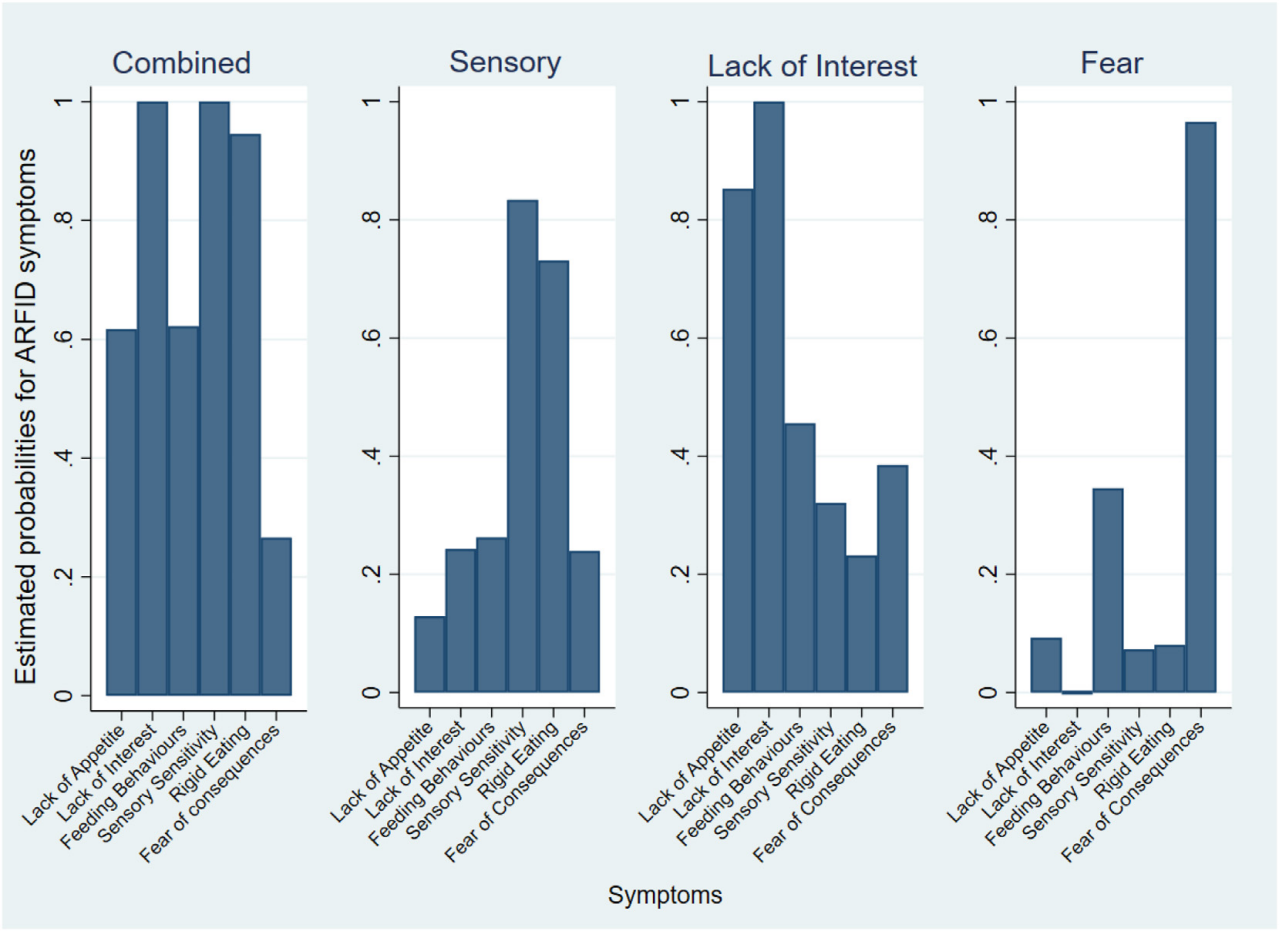
Estimated item-response probabilities for ARFID symptoms by each latent class.

Fit indices for the different class models are shown in Table 3. The most parsimonious model was a 4-class model. The estimated probability for ARFID symptoms for all four classes is presented in Fig. 2. Identified classes were labelled as *Combined subtype*, *Sensory subtype*, *Lack of Interest subtype*, and *Fear subtype*. The *Combined subtype* was characterised by high probabilities of all symptoms (0.62 <probability ≤1) except for fear of aversive consequences of eating (0.27). The *Sensory subtype* was characterised by high probabilities of sensory sensitivity (0.83) and rigid eating behaviours (0.73) and low probabilities of the other symptoms (0.13 <probability ≤0.26). The *Lack of Interest subtype* was characterised by high probabilities of lack of appetite (0.85) and lack of interest in eating or food (1.0), medium-sized probability of difficulties with practicalities of feeding behaviour (0.46) and low probabilities of the other symptoms (0.23 <probability ≤0.39). The *Fear subtype* was characterised by high probability of fear of aversive consequences of eating (0.97) and low probabilities of all other symptoms (0.0 <probability ≤0.35). Overall, 38.2% (*n* = 122) of individuals were classified into the *Combined subtype*, 29.5% (*n* = 94) into the *Sensory subtype*, 25.1% (*n* = 80) into the *Lack of Interest subtype*, and 7.2% (*n* = 23) into the *Fear subtype*.

**Table 3:** Model comparison table of fit statistics for latent class models.

	One class	Two classes	Three classes	Four classes	Five classes	Six classes
Log-likelihood	−1248.617	−1195.014	−1159.572	−1137.449	−1131.940	−1127.268
No. of parameters	6	13	20	27	34	41
Chi square goodness-of-fit tests						
Degrees of freedom	57	50	43	36	29	22
LRT χ^2^	262.320	155.116	84.231	39.986	28.968	19.622
*p* value	<0.001	<0.001	<0.001	0.298	0.4668	0.607
Pearson χ^2^	347.848	181.546	96.765	39.463	27.390	15.601
*p* value	<0.001	<0.001	<0.001	0.318	0.550	0.8352
Bivariate Pearson chi-square	200.904	97.264	16.132	2.699	0.647	0.347
Bivariate log-likelihood chi-square	208.743	97.095	16.261	2.695	0.647	0.347
Number of significant standardized residuals	12	6	0	0	0	0
Information criterion						
AIC	2509.233	2416.029	2359.143	2328.899	2331.881	2336.535
BIC	2531.824	2464.976	2434.447	2430.559	2459.897	2490.908
Sample-size-adjusted BIC	2512.794	2423.743	2371.011	2344.920	2352.056	2360.864
Entropy		1.000	0.754	0.766	0.775	0.752
Model comparisons (T + 1 classes vs. T classes)						
Difference in no. of parameters		7	7	7	7	7
LMR adjusted LRT value		104.612	69.171	43.175	10.752	9.119
LMR adjusted LRT *p* value		<0.001	<0.001	0.020	0.200	0.210
Bootstrap LRT *p* value		<0.001	<0.001	<0.001	0.667	0.600

AIC = Akaike Information Criterion; BIC = Bayesian Information Criterion; LMR = Lo-Mendell-Rubin; LRT = likelihood ratio test.

Average posterior probabilities for the assignment to classes *Combined subtype*, *Sensory subtype*, *Lack of Interest subtype*, and *Fear subtype* were 0.81, 0.91, 0.90 and 0.94 respectively, suggesting low classification error.

### Characteristics of the identified classes

ANOVA and χ^2^ tests were used to explore significant differences between classes and are shown in Table 4. Observed differences across the *Combined subtype*, *Sensory subtype*, *Lack of Interest subtype*, and *Fear subtype* were noted for the following variables: age at diagnosis (F [3, 315] = 14.75, *p* < 0.001); sex (χ^2^ [3, N = 319] = 35.78, *p* < 0.001); duration of symptoms (F [3, 268] = 6.12, *p* < 0.001); duration of symptoms longer than 1 year (χ^2^ [3, N = 272] = 28.46, *p* < 0.001); standard deviation score (SDS) weight (F [3, 240] = 8.12, *p* < 0.001); BMI (F [3, 235] = 2.89, *p* = 0.036); SDS BMI (F [3, 235] = 12.54, *p* < 0.001); weight loss (χ^2^ [3, N = 319] = 40.71, *p* < 0.001); absence of food groups (χ^2^ [3, N = 317] = 43.40, *p* < 0.001); nutritional deficiency (χ^2^ [3, N = 319] = 21.06, *p* < 0.001); reliant on nutritional supplements (χ^2^ [3, N = 318] = 11.30, *p* = 0.010); distress with eating (χ^2^ [3, N = 319] = 9.00, *p* = 0.029); inability to eat with others (χ^2^ [3, N = 319] = 8.44, *p* = 0.038); avoidance of social activities involving food (χ^2^ [3, N = 319] = 10.32, *p* = 0.016); anxiety (χ^2^ [3, N = 263] = 9.10, *p* < 0.028); learning disabilities (LD) (χ^2^ [3, N = 276] = 20.06, *p* < 0.001); and ASD (χ^2^ [3, N = 292] = 38.09, *p* < 0.001).

**Table 4:** Demographics, anthropometrics, clinical characteristics, and comorbidities of the identified latent classes.

	Combined (n = 122)	Sensory (n = 94)	Lack of interest (n = 80)	Fear (n = 23)	Overall *p* value	ɳ^2^/φ_c_
Mean age at diagnosis (SD)	9.9 (3.7)^a^	11.0 (3.6)^a^	13.0 (3.5)^b^	12.9 (2.8)^a,b^	<0.001	0.12
Female n (%)	32 (26.2)^a^	47 (50.0)^b^	48 (60.0)^b^	18 (78.3)^b^	<0.001	0.33
Duration of symptoms in years (SD)	4.8 (3.6)^a^	4.4 (3.9)^a^	3.7 (4.3)^a^	1.0 (1.5)^b^	<0.001	0.06
Duration of symptoms (>1 year) n (%)	85 (81.7)^a^	57 (74.0)^a,b^	45 (63.4)^b^	5 (25.0)^c^	<0.001	0.32
SDS height (SD)	−0.21 (1.40)	−0.27 (1.29)	−0.44 (1.46)	−0.26 (1.21)	0.798	0.00
SDS weight (SD)	−0.64 (1.62)^a^	−0.79 (1.71)^a^	−1.79 (1.70)^b^	−1.75 (1.31)^a,b^	<0.001	0.09
BMI (SD)	15.96 (2.39)^a^	16.55 (3.42)^a^	15.59 (3.10)^a^	14.49 (2.21)^b^	0.036	0.04
SDS BMI (SD)	−0.77 (1.57)^a^	−0.89 (1.89)^a^	−2.06 (1.66)^b^	−2.62 (1.37)^b^	<0.001	0.14
Weight loss n (%)	72 (59.0)^a^	51 (54.2)^a^	74 (92.5)^b^	21 (91.3)^b^	<0.001	0.36
Absence of food groups n (%)	92 (75.4)^a^	45 (48.9)^b^	31 (38.8)^b,c^	4 (17.4)^c^	<0.001	0.37
Nutritional deficiency n (%)	72 (59.0)^a^	40 (42.6)^a,b^	25 (31.3)^b^	5 (21.8)^b^	<0.001	0.26
Tube feeding n (%)	12 (9.8)	11 (11.7)	9 (11.3)	2 (8.7)	0.957	0.03
Reliant on nutritional supplements n (%)	66 (54.6)^a^	34 (36.2)^b^	32 (40.0)^a,b^	6 (26.1)^a,b^	0.010	0.19
Distress with eating n (%)	98 (80.3)^a^	62 (66.0)^a^	51 (63.8)^a^	18 (78.3)^a^	0.029	0.17
Inability to eat with others n (%)	70 (57.4)^a^	41 (43.6)^a^	35 (43.8)^a^	7 (30.4)^a^	0.038	0.16
Avoidance of social activities with food n (%)	81 (66.4)^a^	43 (45.7)^b^	42 (52.5)^a,b^	11 (47.8)^a,b^	0.016	0.18
Depression n (%)	5 (5.5)	5 (6.9)	13 (17.1)	2 (9.5)	0.064	0.17
Anxiety n (%)	45 (47.9)^a^	42 (56.0)^a,b^	37 (49.3)^a^	16 (84.2)^b^	0.028	0.19
OCD n (%)	3 (3.5)	9 (12.5)	3 (4.1)	2 (9.5)	0.092	0.16
DSH n (%)	4 (4.5)	4 (5.9)	7 (9.6)	0 (0.0)	0.337	0.12
LD n (%)	37 (37.0)^a^	17 (21.5)^a,b^	11 (14.3)^b^	0 (0.0)^b^	<0.001	0.27
ADHD n (%)	12 (12.4)	6 (7.8)	4 (5.2)	1 (4.8)	0.335	0.11
ASD n (%)	70 (66.0)^a^	46 (52.9)^a^	21 (26.9)^b^	3 (14.3)^b^	<0.001	0.36

Within each row, different letter superscripts in columns indicate a significant pairwise group difference (*p* < 0.05) and common superscripts indicate no significant group difference (*p* > 0.05) in the post-hoc pairwise comparisons. For example, superscripts a and b in two columns indicate a significant difference. Where there are no superscripts in a row this indicates pairwise comparisons were not conducted as the overall *p*-value was not significant (*p* > 0.05).

ADHD = attention deficit hyperactivity disorder; ASD = autism spectrum disorder; BMI = body mass index; DSH = deliberate self-harm; LD = learning difficulties; OCD = obsessive compulsive disorder; SD = standard deviation; SDS = standard deviation score.

Post-hoc tests with Bonferroni correction were applied and adjusted residuals were calculated to examine pairwise differences, shown in Table 4 with different superscripts indicating group differences. Post-hoc test revealed that individuals in the *Lack of Interest* subtype were significantly older than the ones in the *Combined subtype* and the *Sensory subtype* (*p* < 0.001) and that significantly more boys were classified into the *Combined subtype* than in the other three classes (*p* < 0.001). Individuals in the *Fear subtype* had a significantly shorter duration of symptoms than the ones in the other three classes (*p* < 0.001). Individuals classified into the *Lack of Interest* subtype had significantly lower SDS weight than the ones in the *Combined subtype* and the *Sensory subtype* (*p* < 0.001). Individuals in the *Fear subtype* had significantly lower BMI than the ones in the other three classes (*p* = 0.036). The *Fear subtype* and the *Lack of Interest* subtype had significantly lower SDS BMI and more reported weight loss than the other two (*p* < 0.001). Individuals in the *Combined subtype* had significantly more reported absence of food groups than the ones in the other three classes (*p* < 0.001) and more reported nutritional deficiency than those in the *Lack of Interest subtype* and the *Fear subtype* (*p* < 0.001). Individuals classified into the *Combined subtype* were significantly more reliant on nutritional supplements (*p* = 0.010) and avoided more social activities involving food (*p* = 0.016) than those classified into the Sensory subtype. The *Fear subtype* had significantly more anxiety than the *Combined subtype* and the *Lack of Interest subtype* (*p* = 0.028). Individuals classified into the *Combined subtype* had significantly more LD than the *Lack of Interest subtype* and the *Fear subtype* (*p* < 0.001). The *Combined subtype* and the *Sensory subtype* had significantly more ASD than the other two classes (*p* < 0.001).

### Multinomial logistic regression

**Fig. 3:**
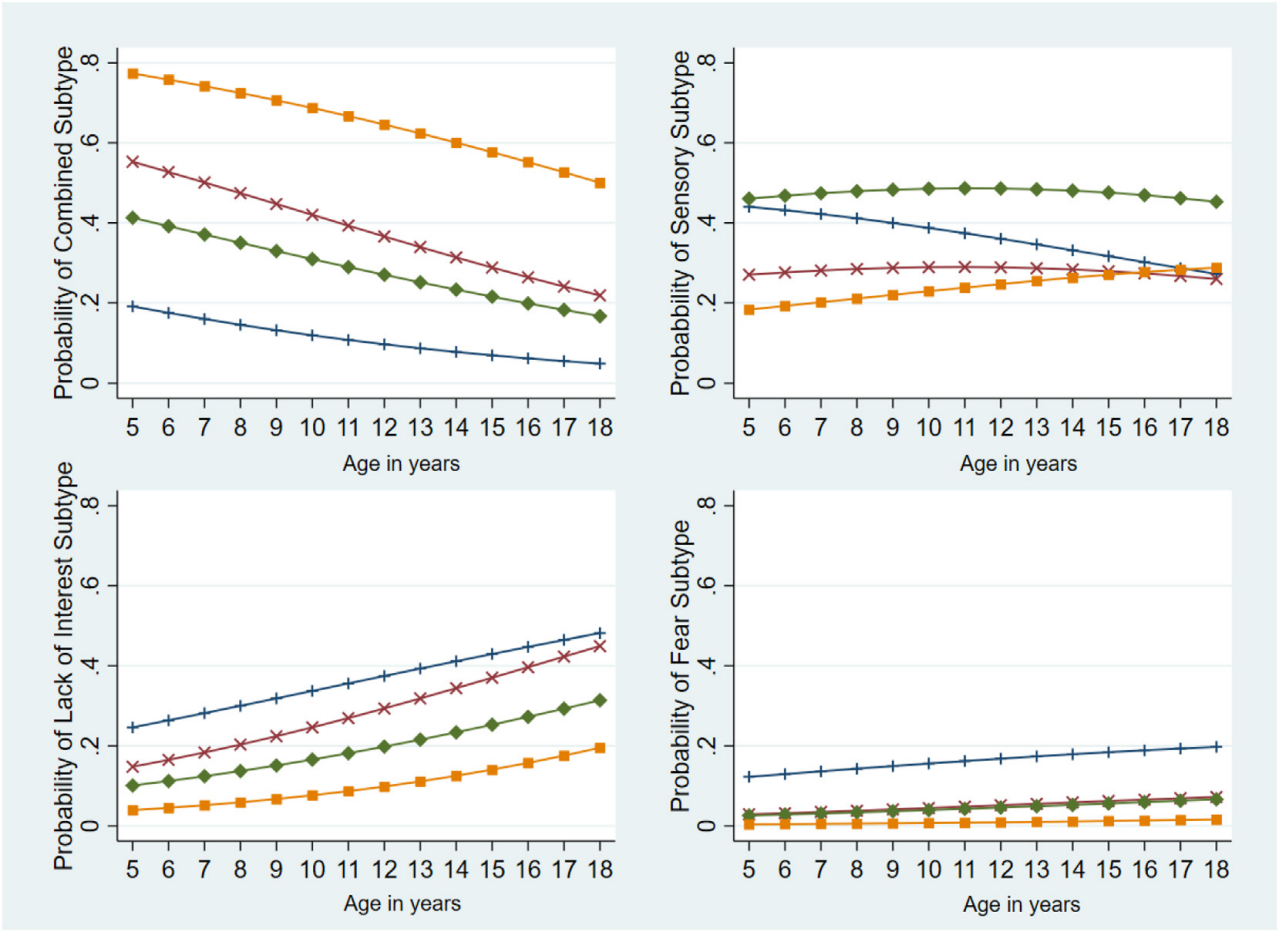
Predicted probabilities of class membership by age, sex, and ASD diagnosis. Figure 3 shows the estimated predicted probabilities of class membership from 5 to 18 years by sex and ASD diagnosis while holding the other two covariates (weight loss and distress with eating) at their means. Orange squares = young males with autism spectrum disorder; red crosses = young males without autism spectrum disorder; green diamonds = young females with autism spectrum disorder; blue crosses = young females without autism spectrum disorder.

MLR was conducted to identify predictors of class membership. A model with age at diagnosis, sex, weight loss, distress associated with eating, and autism spectrum disorder diagnosis (ASD) as covariates was the best fit. Due to missing values, 27 individuals (8.5%) were excluded from the MLR. The *Combined subtype* was treated as the reference category as it was the alternative with most observations. Table 5 shows results of MLR. Individuals in the *Sensory subtype* were less likely to be male (Relative Risk Ratio (RRR) = 0.21; 95% CI [0.11, 0.42], *p* < 0.001), less likely to present with distress associated with eating (RRR = 0.29; 95% CI [0.13, 0.61], *p* = 0.001) and less likely to have a diagnosis of ASD (RRR = 0.48; 95% CI [0.25, 0.93], *p* = 0.030) compared to those in *Combined subtype*. Individuals in the *Lack of Interest subtype* were less likely to be male (RRR = 0.21; 95% CI [0.10, 0.45], *p* < 0.001), less likely to have distress associated with eating (RRR = 0.17; 95% CI [0.07–0.40], *p* < 0.001), less likely to have a diagnosis of ASD (RRR = 0.19; 95% CI [0.09–0.40], *p* < 0.001), and more likely to be older (RRR = 1.17; 95% CI [1.05, 1.30], *p* = 0.003) and have reported weight loss (RRR = 5.09; 95% CI [1.87, 13.89], *p* = 0.001) than participants in the *Combined subtype*. Finally, individuals in the *Fear subtype* were less likely to be male (RRR = 0.08; 95% CI [0.02–0.29], *p* < 0.001), less likely to have distress with eating (RRR = 0.26; 95% CI [0.07–0.94], *p* = 0.040) and less likely to have a diagnosis of ASD (RRR = 0.10; 95% CI [0.03–0.38], *p* = 0.001) compared to those in *Combined subtype*. Fig. 3 shows the estimated predicted probabilities of class membership from 5 to 18 years by sex and ASD diagnosis while holding the other two covariates at their means.

**Table 5:** Multinomial logistic regression predicting class membership relative to the Combined Subtype.

	Sensory vs. combined		Lack of interest vs. combined		Fear vs. combined	
	RRR (95% CI)	*p* value	RRR (95% CI)	*p* value	RRR (95% CI)	*p* value
Sex (male)	0.21 (0.11–0.42)	<0.001	0.21 (0.10–0.45)	<0.001	0.08 (0.02–0.29)	<0.001
Age (year)	1.07 (0.98–1.17)	0.139	1.17 (1.05–1.30)	0.003	1.15 (0.97–1.36)	0.089
Weight loss	0.71 (0.37–1.37)	0.309	5.09 (1.87–13.89)	0.001	3.47 (0.70–17.25)	0.129
Distress with eating	0.29 (0.13–0.61)	0.001	0.17 (0.07–0.40)	<0.001	0.26 (0.07–0.94)	0.040
ASD	0.48 (0.25–0.93)	0.030	0.19 (0.09–0.40)	<0.001	0.10 (0.03–0.38)	0.001

ASD = autism spectrum disorder; CI = confidence interval.

## Discussion

To our knowledge this is the first study using LCA to identify classes of ARFID based on the proposed DSM-5 drivers of food avoidance in a large sample of children and adolescents presenting to secondary clinical care. Cases were reported through national surveillance units which survey most of the consultant paediatricians and child and adolescent psychiatrists in the UK and ROI, rather than from specialty eating disorder services^6^^,^^7^^,^^10^^,^^15^ or paediatricians alone.^18^

A prior study used LCA in a paediatric sample of children and adolescents with ARFID from Canada. Three classes were identified and labelled *Acute Medical*, *Lack of Appetite*, and *Sensory* which they suggested resembled the three proposed DSM-5 ARFID subtypes. However, the first two classes had similar patterns of response to the variable indicators used to define classes and a considerable number of individuals were assigned to more than one class with similar probability. The authors^18^ proposed that these individuals belonged to a combination of those subgroups, suggesting the existence of a fourth subgroup which was not presented as a class.^18^ Our study used six simple binary indicators of proposed drivers of food avoidance to empirically derive ARFID subgroups, which we suggest offers a more robust methodology. Additionally, our study was the first to explore potential predictors of class membership.

Based on fit statistics, LCA suggested that the four-class model was the best. Interpretation of the four-class model broadly supported the existence of three subtypes of ARFID, as originally proposed by Bryant-Waugh et al. and included the DSM-5.^1^ It also has similarities with prior literature suggesting ARFID subtypes are not mutually exclusive.^6^, ^7^, ^8^^,^^10^^,^^15^^,^^31^ Thomas et al. (2017) proposed a dimensional model, where the three subtypes may vary in severity but are not separate diagnostic groups.^31^ This is supported by our results from a national sample, where the most prevalent class, comprising 38% of the sample, was the *Combined Subtype* characterised by a combination of *Lack of Interest Subtype* and *Sensory Subtype*. Other studies have reported that more than 50% of individuals with ARFID have mixed presentations^6^, ^7^, ^8^ with one study reporting that 10% have the three subtypes.^7^ Consistent with our findings, two studies revealed that the most prevalent mixed presentation was the one characterised by selective eating and appetite disturbance.^7^^,^^10^

This study provides new information about how the clinical characteristics of ARFID presentations differ by sex and age. Younger age at diagnosis, being male, experiencing high levels of distress related to eating/food, and having a diagnosis of ASD were predictive of membership in the *Combined Subtype*. Having less reported weight loss was associated with the *Sensory Subtype* with a 50:50 sex distribution. These two subgroups more often had comorbidity with ASD or LD than the other two subgroups. Older age at diagnosis, weight loss, absence of distress around eating, and no diagnosis of ASD were predictive of membership in the *Lack of Interest Subtype*. Lastly, being female and not having a diagnosis of ASD was predictive of membership in the *Fear Subtype* class. Consistent with our findings, prior research has found differences across sexes with one study reporting that male patients with ARFID showed more food avoidance caused by sensory sensitivities than females.^4^ Moreover, patients who present with lack of interest have been associated with weight loss.^10^ However, our results contrast with the recent findings by Watts et al. (2023)^11^ where it was reported that ARFID subtypes do not differ between sexes and that ASD does not lead to a different presentation of ARFID.

Individuals in the *Combined Subtype* or *Sensory Subtype* had mean BMI z-scores in the normal weight range, in line with the fact that clinically significant ARFID occurs across the weight spectrum.^14^ In contrast, those in the other two subtypes presented with underweight, with more than 90% having lost weight according to the responsible clinician. While individuals in the *Fear Subtype* typically have an acute onset, those in the *Lack of Interest Subtype* often have long-standing symptoms, likely due to chronic low appetite associated with decreased activation of the brain’s appetite-regulating centres.^31^

The *Fear Subtype* was the least common, comprising only 7% of this sample. In contrast with our findings, studies using samples exclusively from eating disorders services showed a higher prevalence of the aversive (fear) subtype,^10^^,^^15^^,^^32^ likely due to a low comorbidity with ASD in those samples. Our results show that the *Fear Subtype* had characteristics that differ from the other three subgroups: almost 80% of this subgroup were girls, were underweight, ARFID symptoms had acute onset, and more than 80% had comorbid anxiety. Although we support the idea that ARFID is a dimensional disorder, results from this study suggest that the *Fear Subtype* may be a distinct and mutually exclusive group, lacking mixed characteristics with the other presentations. Moreover, this group, with a more acute onset, may present to different services from the other subtypes.

Clinicians should be aware of the heterogeneity of this disorder as different presentations may benefit from different interventions. Our findings imply the importance of individualised treatment for patients with ARFID. Different psychological treatment models may be effective for different subtypes, depending on the driving psychopathology, and there is evidence to suggest medications may be beneficial for certain groups. Individuals in the *Fear Subtype* have high rates of comorbid anxiety and therefore may benefit from a short trial of anxiolytic medication such as lorazepam.^33^ Patients with the *Lack of Interest Subtype* could benefit from cyproheptadine,^34^ an antihistamine used to stimulate appetite. Regarding the treatment of sensory sensitivities to food characteristics, no medication has proven effective to date. Disgust, a suggested underlying mechanism in those presentations, is reported to be more resistant to extinction than anxiety and interventions developed primarily for the latter may have limited efficacy.^35^ The *Combined Subtype* and the *Sensory Subtype*, may benefit from interventions used to treat children and adolescents with comorbid ASD, learning disabilities, and sensory sensitivities. Future randomised placebo-controlled trials are needed to evaluate the efficacy of pharmacological and psychological interventions in ARFID subtypes.

The aetiology of ARFID is still poorly understood but a recent twin study in Sweden suggested that there is an important genetic contribution with high heritability,^36^ though this dataset did not include analysis of fear of aversive consequences of eating and therefore may not reflect heritability in all subtypes. Although there is substantial overlap at the symptom level between ARFID subtypes, there could be genetic differences between ARFID subtypes.^37^ Collaborative efforts should be undertaken to conduct a genome-wide association study (GWAS) for ARFID that would help clarify its biological mechanisms and improve our understanding of the subtypes^37^ which will help inform novel treatment targets that may improve outcomes for these patients.

Our study benefits from several strengths. This is the largest sample to date of children and adolescents with ARFID. Cases were identified through active surveillance which allows ascertainment of data from all paediatricians and child and adolescent psychiatrists in the UK and ROI. This sample includes young people of different ages and geographical areas, ensuring data is representative of those presenting for clinical care in medical settings. We suggest our study improves on the first attempt to classify children and adolescents with ARFID using LCA, by using only symptoms related to ARFID phenomenology, with a more robust methodology, and by identifying predictors of class membership.

Nevertheless, a few limitations warrant consideration. Surveillance methodology is limited to cases seen in teams that have a consultant paediatrician or child and adolescent psychiatrist, so ARFID cases treated in primary care or in psychology services will not be included. Case ascertainment in surveillance studies relies on clinicians’ ability to make accurate diagnoses. In recent years, clinicians have become more familiar with ARFID, but diagnostic challenges still exist. For example, children with ASD often present with feeding difficulties and identifying those that reach the diagnostic threshold for ARFID may be difficult. In other studies, the prevalence of ASD among children with ARFID ranges widely, with estimates from 8.2% to 54.75%,^3^ so while we did see a high level of comorbidity of ARFID with ASD, this does not necessarily represent diagnostic bias. Another limitation is that ARFID data were collected using a questionnaire developed by the authors and diagnoses were not validated using a clinical instrument such as the Pica, ARFID, and Rumination Disorder Interview (PARDI).^38^

We focused on children and adolescents; in the future, studies with adult samples will help to better understand this disorder. In our study we used indicator variables based on the psychopathology of ARFID but future studies should investigate other ARFID subtypes not yet considered such as a gastrointestinal subtype^39^ or a somatically-focused subtype.^40^

Due to sample size and missing data only 5 covariates were included in MLR which limits the ability to identify other predictors. In order to maximise response rate, the questionnaire responses were not mandatory, therefore clinicians could fill is as much or as little information as they knew. As long as there was enough information to confirm a case of ARFID, the case was included. In the future, analyses should be undertaken in larger samples ascertained through international collaboration.

In conclusion, this study aimed to explore extant subgroups in a large sample of children and adolescents with ARFID presenting to secondary care in the UK and ROI. Using LCA, four subgroups were identified. The commonest class was the *Combined Subtype*, a combination of the *Lack of Interest subtype* and the *Sensory subtype*. The other three classes represent subtypes proposed by the DSM-5. The *Fear subtype* was the least common and exhibited distinct characteristics, suggesting it may be a separate category. Predictors of membership in each class were also explored, revealing different associations with age, sex, weight loss, distress associated with food, and autism diagnosis. Clinicians should be aware of these characteristics as they can influence treatment and decision-making.

## Contributors

DN contributed to the conception of the study. DN, JN, RML, and LH contributed to the design and development of the study. JSC contributed to the development of the study, carried out the analysis, and wrote the first draft of the article with contributions from NJ. JSC and NJ collected the data. JSC, JN, and DN accessed and verified the data underlying the study. TC reviewed and verified the analysis. All authors edited and approved the final version of the Article. All authors confirmed that they had full access to all the data in the study and accept responsibility to submit for publication.

## Data sharing statement

Deidentified participant data that underlie the results reported in this Article can be shared upon specific requests by researchers who provide a methodologically sound proposal. The proposal will be considered by the investigators of this Article. Requests should be directed by email to the corresponding author.

## Declaration of interests

In the past three years, JSC declares a grant funded by the Fundación Alicia Koplowitz. In the past three years, DN declares grants or contracts from North West London NIHR applied research collaboration; NIHR SD&O; Rosetrees Foundation; Imperial College BRC; HQIP; Fundación Alicia Koplowitz; and NHS England. DN has received royalties from Springer. DN has received honoraria for contribution from MindEd; and the British Association of Psychopharmacology. DN has a leadership role in BEAT (Charity) Clinical Advisory Group and as a National Clinical Advisor in NHS England. All other authors have no interest to declare.
